# Waveguiding of Photoluminescence in a Layer of Semiconductor Nanoparticles

**DOI:** 10.3390/nano11030683

**Published:** 2021-03-09

**Authors:** Yera Y. Ussembayev, Natalia K. Zawacka, Filip Strubbe, Zeger Hens, Kristiaan Neyts

**Affiliations:** 1 LCP Research Group, Ghent University, Technologiepark 126, 9052 Gent, Belgium; Yerzhan.Ussembayev@UGent.be (Y.Y.U.); NataliaKlaudia.Zawacka@UGent.be (N.K.Z.); Filip.Strubbe@UGent.be (F.S.); 2 Center for Nano and Biophotonics, Ghent University, Technologiepark 126, 9052 Gent, Belgium; Zeger.Hens@UGent.be; 3 PCN Research Group, Ghent University, Krijgslaan 281, 9000 Gent, Belgium

**Keywords:** nanooptics, anisotropic emission, light waveguiding, quantum dots, LED, solar concentrators

## Abstract

Semiconductor nanoparticles (SNPs), such as quantum dots (QDs) and core/shell nanoparticles, have proven to be promising candidates for the development of next-generation technologies, including light-emitting diodes (LEDs), liquid crystal displays (LCDs) and solar concentrators. Typically, these applications use a sub-micrometer-thick film of SNPs to realize photoluminescence. However, our current knowledge on how this thin SNP layer affects the optical efficiency remains incomplete. In this work, we demonstrate how the thickness of the photoluminescent layer governs the direction of the emitted light. Our theoretical and experimental results show that the emission is fully outcoupled for sufficiently thin films (monolayer of SNPs), whereas for larger thicknesses (larger than one tenth of the wavelength) an important contribution propagates along the film that acts as a planar waveguide. These findings serve as a guideline for the smart design of diverse QD-based systems, ranging from LEDs, where thinner layers of SNPs maximize the light outcoupling, to luminescent solar concentrators, where a thicker layer of SNPs will boost the efficiency of light concentration.

## 1. Introduction

A growing demand for sustainable energy development stimulates the ongoing search for new materials and engineering designs that will improve the performance of modern technologies. Semiconductor nanoparticles (SNPs) are attractive nanomaterials that could help to diminish the energy losses and maximize the efficiency of different devices, including light-emitting diodes (LEDs) [1], displays [2,3] and solar concentrators [4]. These light-emitting technologies usually require the deposition of a thin layer of colloidal SNPs on a substrate in order to deploy their optical and electrical properties efficiently. For instance, present quantum dot (QD)-based displays and LEDs have a multilayered structure of SNPs with thicknesses in the order of tens of nanometers, which enables bright and color-saturated emission [5]. Other QD applications, such as solar concentrators, utilize SNPs as sunlight collectors suspended in a polymer slab, which waveguides the down-converted light towards the solar cells by total internal reflection (TIR). However, the efficiency of all these light-generating systems still suffers from uncontrollable light outcoupling or waveguiding. Particularly, for LEDs and displays this may result in up to 50–95% photon losses, due to reduced light outcoupling, undesirable waveguiding or coupling to surface plasmon modes near metal electrodes [1]. In addition, for solar concentrators based on spherical QDs, the maximum attainable fraction of guided emission may achieve only 74% due to photons outcoupling out of the TIR cone angle [6]. Therefore, there is a growing need to understand the underlying mechanisms that govern the direction of the emitted light in order to enhance the efficiency of QD-based devices. 

In this work, we aim to obtain deeper insights as to how the thickness of SNP film impacts the outcoupling and waveguiding of the emitted light. Over the past few decades, models have been developed to study nanostructured systems, following the approaches of Maxwell-Garnett or Bruggeman [7,8,9]. These methods describe a complex structure by introducing an equivalent homogeneous medium, which leads to an effective dielectric constant *ε_eff_*. These theoretical frameworks can be used effectively to study light absorption or scattering by this medium [10]. However, these approaches are appropriate when the volume fraction of nanoparticles is small, and important corrections are necessary when the fraction increases above 20%. In this work we consider the case of a single layer or a multilayer of nanoparticles in which the volume fraction of particles can be significantly higher than 20%. This case is very relevant, because nanoparticles are often deposited in multilayers with high packing density. We first evaluate the magnitude of the dielectric depolarization effect and its effect on the anisotropy for absorption by layered SNPs. We then carry out detailed static field calculations and use these to obtain the effective thickness *d_eff_* of an equivalent layer of homogeneous material. The latter parameter is then used to calculate the emission from a dipole emitter embedded in a QD layer as proposed in some reports [11,12,13,14]. In Section 3 we show how the effective thickness allows estimation of the effect of the film thickness on the light outcoupling and waveguiding. The main result of this work is that monolayers, or very thin layers of QDs, lead to negligible waveguiding and most of the photoluminescent emission is outcoupled, while thicker layers lead to stronger waveguiding. To verify the validity of our proposed model, we provide a set of fluorescence microscopy experiments for very thin layers and for somewhat thicker layers of CdSe/CdS core/shell nanocrystals. Finally, we also discuss how the thickness of a QD film can improve the efficiency of light-generating devices, such as LEDs, displays and solar concentrators.

## 2. Materials and Methods

### 2.1. Analytical Expressions 

The full analytical framework can be found in the Supplementary Materials.

### 2.2. Static Field Calculations of Absorption and Effective Thickness

To calculate the absorption properties of a stack of SNPs, we applied the quasistatic approximation based on COMSOL Multiphysics 5.3 (Electrostatics module). For the squared or triangular packing of SNPs, a spherical particle with dielectric constant *ε_p_* = 6*ε_o_* (CdS [15] for a free space wavelength *λ_o_* = 500 nm) was enclosed within a 3D square or hexagonal prism unit cell (Figure S1a) with dielectric constant of the surrounding medium *ε_m_*, which varied between *ε_o_* and 6*ε_o_*. To simulate the interaction of a homogeneous external electric field *E_e_* with an infinitely extended SNP layer, we solved the static potential problem ∇(*ε⋅*∇*V*) = 0 with appropriate boundary conditions. For the faces parallel to the external field, Neumann boundary conditions were used. For the faces perpendicular to the external field, a Dirichlet boundary condition with constant voltage was used. For faces that made an angle with the external field, the Dirichlet condition *V*(*r*) = −*E_e_*∙*r* was used. The tetrahedral mesh of the geometry (Figure S1b) was generated with a maximum element size *d_SNP_*/15 and a maximum element growth factor of 1.25. The values *β_ij_*(*r*) were calculated as a ratio of the *i*th component of the internal electric field *E_i_*(*r*) over the amplitude of the applied external field *E_j_*. The obtained values were used to calculate the absorption anisotropy *a_σ_* and the effective thickness *d_eff_* according to the Equations (2) and (6), respectively.

### 2.3. Full-Wave Simulations of Emission Outcoupling and Waveguiding 

To simulate the wave propagation within the layer of SNPs, we employed the commercial full-wave finite-element solver COMSOL Multiphysics 5.3 (Wave Optics module). The designed 2D model geometry and corresponding mesh are shown in Figure S1. The stack of semiconductor nanowires (aligned towards the viewer) or equivalent slab with effective thickness *d_eff_* were embedded inside the dielectric medium enclosed with perfectly matched layers (PMLs), which absorbed the outgoing radiation. The emission of the quantum dot was modelled as a point dipole emitter (PDE) placed in the center of the geometry and oriented out of the geometry plane. The entire geometry was meshed with triangular elements, which had a maximum element size of *d_SNP_*/12 and element growth rate 1.3. The angular emission patterns in the far field were calculated at the far-field boundary (FFB) and normalized to the maximum field amplitude obtained for the considered examples.

### 2.4. Synthesis of Core/Shell Nanoparticles

CdSe/CdS core/shell SNPs were synthesized by adapting previously reported procedures [16,17,18], consisting of CdSe (core) quantum dots and subsequent growth of CdS (shell) material surrounding the initial particle. CdSe wurtzite particles were synthesized starting from CdO (≥99.99%, Sigma-Aldrich, Munich, Germany), N-tetradecylphosphonic acid (TDPA, ≥97%, PlasmaChem GmbH, Berlin, Germany), oleyl alcohol (OlOH, 85%, Sigma-Aldrich, Munich, Germany) and 10 g of trioctylphosphine oxide (TOPO, Merck Millipore, Darmstadt, Germany), molar ratio Cd:TDPA:OlOH 1:6:16. The reaction mixture was heated for 1 h at 150 °C under a nitrogen atmosphere. The solution was then heated to 350 °C in order to dissolve CdO, then 2 mL of trioctylphosphine (TOP) was injected, followed by the injection of 2 M TOP-Se solution, Cd:Se molar ratio 1:2. The reaction time was limited to a few seconds and stopped by a drop in temperature. CdSe/CdS wurtzite particles were synthesized from CdO, oleic acid and 5 g of trioctylphosphine oxide; Cd:oleic acid molar ratio 1:5. The reaction mixture was heated for 1 h at 150 °C under a nitrogen atmosphere. The solution was then heated to 350 °C in order to dissolve CdO, then 1 mL of trioctylphosphine was injected, followed by the injection of 2 mL of reaction solution. The reaction solution consisted of previously synthesized QD seeds CdSe, 2.4 M TOP-S solution and additional TOP solvent; Cd:S molar ratio 1:1.2. After 5 min the reaction was quenched by a drop in temperature and the particles were precipitated with 10 mL of methanol. The QDs were dispersed and stored in toluene. The quantum efficiency of the QD solution was determined by an integrating sphere analysis via the two-measurement approach [19], yielding 65%. 

### 2.5. Fabrication and Characterization of SNP Films

**Figure 1:**
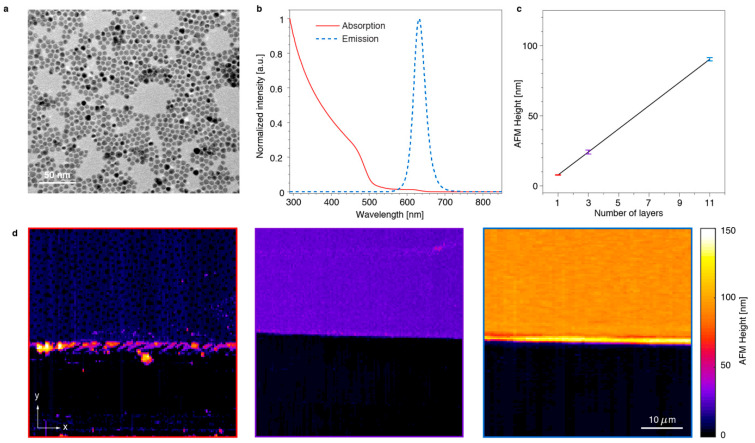
Fabrication and characterization of CdSe/CdS core/shell nanocrystals and thin films. Transmission electron microscopy image (**a**), absorption and emission spectra (**b**) of the synthesized CdSe/CdS nanoparticles. Atomic force microscopy (AFM) measurements of the thickness of the layered semiconductor nanoparticle (SNP) samples (**c**) with corresponding images (**d**) of a monolayer (left), three layers (middle) and eleven layers (right) of quantum dots.

The size of the QDs was determined by transmission electron microscopy (TEM) analysis, giving a diameter of 7.4 nm (+/− 0.8 nm) for CdSe/CdS (Figure 1a). Bright field TEM images were taken using a Cs corrected JEOL-200FS microscope (JEOL, Tokyo, Japan). Absorption spectra (Figure 1b) were taken using a spectrometer (Lambda 950, Perkin Elmer, Waltham, MA, USA). Spin coating was used to provide uniformly distributed layers of QDs. Thin film samples were prepared from QD colloidal dispersions on pre-cleaned rectangular microcovered glass substrates (VWR, Radnor, PA, USA) at a speed of 3000 rpm for 60 s. A stock solution of QDs in toluene was used to prepare different dilutions ranging from 5 to 100 µM in order to obtain films with varying thickness (Figure 1c,d).

### 2.6. Microscopy Imaging of the Samples

**Figure 2:**
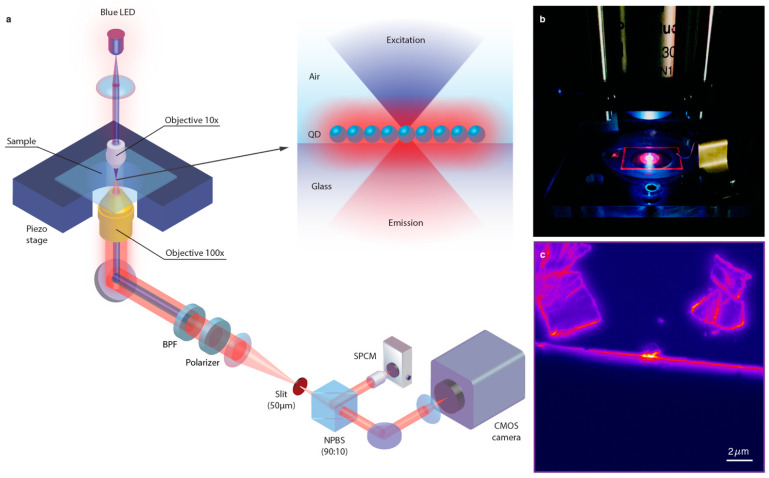
Optical microscopy setup. (**a**) Schematic illustration of the developed setup (not to scale), where the sample is placed on a piezostage and illuminated with a blue light-emitting diode (LED). The emitted light is collected with a 100× objective lens, filtered with a band-pass filter (BPF) and divided by a non-polarizing beam splitter (NPBS) between a single-photon counting module (SPCM) and a complementary metal oxide semiconductor (CMOS) imaging camera; (**b**) photograph of the sample in the setup; (**c**) fluorescence image of the sample.

The samples were visualized with a custom-built fluorescence microscope shown in Figure 2a. A blue LED (C503B-BCS-CV0Z0461, Durham, NC, USA) with a peak wavelength of 470 nm was used as an excitation source (power *P_out_* = 265 μW) and focused with a 10× objective lens (Plan Fluor, NA 0.30, Nikon, Tokyo, Japan) on the sample placed on a piezoactuator stage (Tritor 102SG, PiezoSystemJena, Jena, Germany). The QD emission was collected with a 100× oil-immersion objective lens (Plan Fluor, NA 1.30, Nikon, Japan, Tokyo) from the substrate side and split between a complementary metal oxide semiconductor (CMOS) camera (Andor Zyla 4.2, sCMOS, Oxford Instruments, Oxford, UK) with an acquisition rate of 10 Hz and a single-photon counting module (SPCM-ARQH-15, Excelitas Technologies, Wiesbaden, Germany) with an integration time of 10 ms. The excitation light was blocked with a band-pass filter (ET542lp, Chroma, Bellows Falls, VT, USA) while the emission was integrated over the area of a slit (50 μm × 1 mm, S50RD, Thorlabs, Newton, NJ, USA), aligned parallel to the film edge. The acquired photon counts were averaged with a sliding bin size of 250 ms.

## 3. Results and Discussion

The goal of this work is the analysis of light emission from a layer of semiconductor nanoparticles. As there is reciprocity between absorption and emission, we first investigate the absorption of light in a monolayer of spherical SNPs with diameter *d_SNP_* arranged in a regular square or triangular grid. There can be a distance *d_gap_* between two particles and the grid side is given by the sum *d = d_SNP_ + d_gap_ << λ_ο_*, where *λ_ο_* is a free-space wavelength of light. Typically, SNPs have a dielectric constant *ε_p_* that is much larger than that of the embedding medium *ε_m_ = ε_o_ · n_m_*^2^. When the layer of SNPs is placed in a static homogeneous external field *E_e_*, the field inside the SNPs is typically reduced in amplitude due to dielectric depolarization and the internal field is determined by the position-dependent tensor *β*: ${Ε}_{i}\left(r\right)\;=\;\bar{\bar{\beta }}\left(r\right){Ε}_{e}$. When the incident light has a wavelength much longer than the grid side *d*, the static field approximation can be used to estimate the optical absorption. The absorption is proportional to |*E_i_*(*r*)|^2^ when the dielectric constant of the nanoparticles has a small complex component [6]. When, for example, an external field is applied along the z-axis, perpendicular to the particle layer, then the ratio of the internal squared field |*E_i_*(*r*)|^2^ over the external squared field |*E_e_*|^2^ is given by

(1) $$\beta_{z}^{2}\left(r\right)=\beta_{xz}^{2}\left(r\right)+\beta_{yz}^{2}\left(r\right)+\beta_{zz}^{2}\left(r\right)$$

This quantity, which depends on the ratio of dielectric constants and on the interparticle distance, is proportional to the local absorption in the nanoparticle layer. 

**Figure 3:**
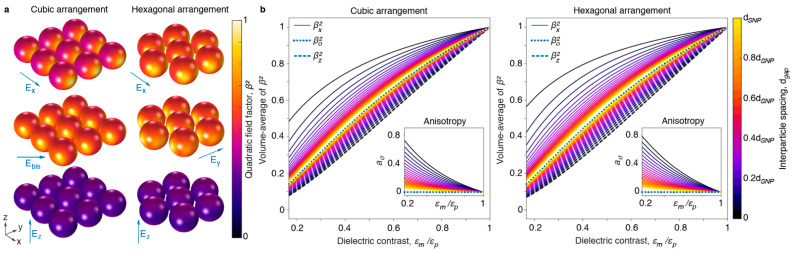
Simulated absorption properties of SNPs arranged in a monolayer. (**a**) Distribution of the quadratic field factor *β*^2^(*r*) for different arrangements of particles with the external field (blue arrow) perpendicular or parallel to the plane of the layer of SNPs, in the medium with *ε_m_* = 2.25*ε_o_* (*n* = 1.5). (**b**) Volume-average <*β*^2^> for SNPs arranged in a square (left) and triangular lattice (right) as a function of dielectric contrast *ε_m_*/*ε_p_* and interparticle spacing *d_gap_*. Inset graphs show the corresponding absorption anisotropy *a_σ_*.

Figure 3a illustrates the spatial dependency of the factor *β*^2^ in the layer of spherical nanoparticles for different directions of the electric field vector of the external incident light. In the closely packed square lattice the incident field is *E_z_*, *E_x_* or along the bisector *E_bis_*. For the triangular grid, the incident field is along *E_z_*, *E_x_* or *E_y_*. In the example shown, the dielectric constants of the embedding medium and the SNPs are 2.25 × *ε_o_* and 6 × *ε_o_*, respectively, which yields *ε_m_/ε_p_* = 0.375. The simulations show that, similar to a planar layer, the field in the nanoparticles is smaller when the external field is perpendicular to the layer. To determine the average absorption in the particle, the quantity *β*^2^ is averaged over the volume of the particle, for the different directions of the incident field. The result in Figure 3b shows the average *<β*^2^*>* as a function of the ratio of the dielectric constants and the interparticle distance *d_gap_*. When *d_gap_* is much larger than *d_SNP_*, the particles are isolated and the total absorption is similar to that of an isolated SNP, with corresponding factor $<\beta_{0}^{2}>$. The ratio between $<\beta_{x}^{2}>$ and $<\beta_{0}^{2}>$ can be considered as the enhancement factor due to incorporation in a layer and has also been studied elsewhere [20], for materials in which the dielectric constant has a significant imaginary part. Note that for in-plane fields, the absorption is independent of the azimuthal angle, due to the four-fold or six-fold symmetry axis of rotation for the structure. Absorption is larger for fields parallel to the layer and smaller for fields perpendicular to the layer. In the following we use the quantities $<\beta_{∥}^{2}>$ for in-plane fields and $<\beta_{⊥}^{2}>$ for perpendicular fields. The anisotropy in the absorption [6] for ∥ versus ⊥ polarized light is defined as *a_σ_*:

(2) $$a_{\sigma }=\frac{\langle \beta_{∥}^{2}\rangle -\langle \beta_{⊥}^{2}\rangle }{\langle \beta_{∥}^{2}\rangle +\langle \beta_{⊥}^{2}\rangle }$$

The anisotropy in absorption is larger when the layer is more densely packed and for larger dielectric contrasts (Figure 3b, insets).

To estimate the light emission from a nanoparticle, we assume that each nanoparticle is well described by an elementary electrical dipole oscillator. Since the particle diameter *d_SNP_* and the gap *d_gap_* are much smaller than the wavelength of the emitted light, quasistatic electric field calculations can be used to estimate the field in the neighborhood of the layer. When the dipole moment of the emitter inside the nanoparticle is *p_i_*, then the field well above or below the layer is described by an equivalent electrical dipole moment *p_e_* present in the medium with dielectric constant *ε_m_*. According to the reciprocity theorem [21], the external dipole moment *p_e_* and the internal dipole moment *p_i_* at position *r* are linked by the transposed tensor *β*:

(3) $$p_{e}={\bar{\bar{\beta }}}^{T}\left(r\right)p_{i}\left(r\right)$$

The equivalent electrical dipole moment in the embedding medium is typically not parallel to the dipole in the internal medium, because the tensor components are different, for example for a layer of SNPs the coefficient *β_zz_* is typically smaller than *β_xx_*.

The radiation of an oscillating dipole *p_i_* in an SNP in a given structure is equal to the radiation of the equivalent oscillating dipole *p_e_* in medium *ε_m_* according to Equation (3). The excited state in the layer of SNPs can return to the ground state by an electrical dipole transition. In ref. [6] it was shown that the assumption of a random orientation of the dipole orientation seems appropriate. In this work we assume that the excitation can happen from any location in the layer. This means that the power emitted from the oscillating electrical dipole *p_i_* should be averaged over all possible orientations and over all positions in the layer *r*. The emission from oscillating dipoles in the SNP layer has three contributions: emission towards the upper medium, emission towards the lower medium and emission that is waveguided by the layer. The emission can have TM and TE contributions. As we assume that the layer is very thin, phase delays can be neglected and the emissions of the dipole *p_e_* towards the upper and lower medium are equal. In the following we investigate the influence of the SNP arrangement on the different contributions.

The power emitted per unit solid angle in the embedding medium *m* for a layer of SNPs that is excited is obtained after averaging the emission of the oscillating dipole *p_i_* over orientations and locations. The TM and TE contributions are, respectively, given by:

(4) $$P_{RND}^{TM}=\frac{\omega^{4}n_{m}p_{i}^{2}}{12\pi \varepsilon_{0}c^{3}}\frac{1}{8\pi }\left[\langle \beta_{⊥}^{2}\rangle \sin^{2}\theta +\langle \beta_{∥}^{2}\rangle \cos^{2}\theta \right]$$

(5) $$P_{RND}^{TE}=\frac{\omega^{4}n_{m}p_{i}^{2}}{12\pi c^{3}\varepsilon_{0}}\frac{1}{8\pi }\langle \beta_{∥}^{2}\rangle$$

In these formulas the *β*^2^ factors are the same as defined previously. As the parallel factor is larger than the perpendicular factor (Figure 3), the TM emission is not isotropic but has a maximum in the direction perpendicular to the layer plane. 

**Figure 4:**
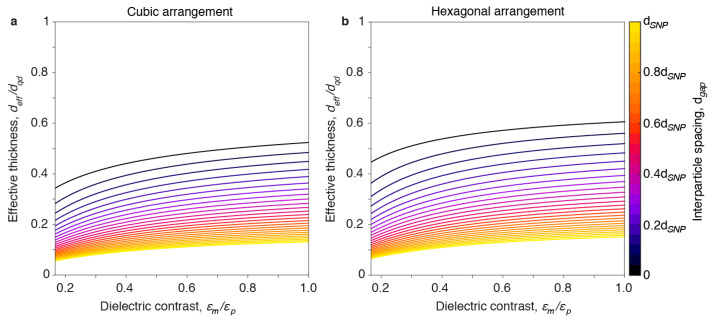
Simulated effective thickness of SNPs arranged in a monolayer. Effective thickness as a function of dielectric contrast *ε_m_*/*ε_p_* and interparticle spacing *d_gap_* for square (**a**) and triangular (**b**) lattice arrangement.

A layer consisting of SNPs is strictly speaking a lossy waveguide, because light propagating along the layer is scattered by the nanoparticles. Here we consider particles with a diameter much smaller than the wavelength of light, such that scattering is limited and we can approximate the layer as a lossless waveguide [11]. When the layer of SNPs is embedded in a medium with a lower refractive index, the *TE*_0_ waveguided mode with an electric field vector parallel to the layer is the most important mode. For a homogeneous slab waveguide with a thickness much smaller than the wavelength of the light, the waveguided *TE*_0_ mode is determined by the difference in dielectric constant and the film thickness (Supplementary Materials). For an inhomogeneous thin film, we assume that the waveguided mode *TE*_0_ is determined by the excess polarization in the layer when a field is applied parallel to the layer. For the case of a *TE*_0_ waveguided mode propagating in the *y*-direction within an inhomogeneous layer consisting of nanoparticles, and with the field mainly polarized in the *x*-direction, the penetration depth in the embedding medium is determined by the effective thickness (Figure 4), which takes into account the excess polarization due to the SNP layer (Supplementary Materials):

(6) $$d_{eff}\approx \frac{1}{S}\int_{V}\frac{n^{2}\left(r\right)\beta_{xx}\left(r\right)-n_{m}^{2}}{n_{p}^{2}-n_{m}^{2}}dV.$$

**Figure 5:**
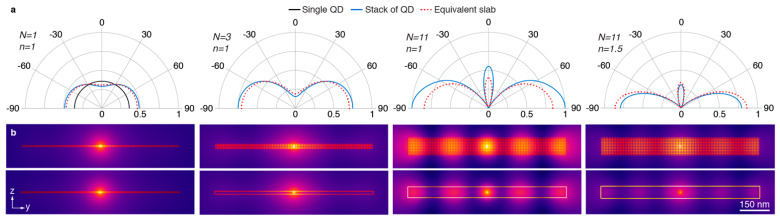
Emission properties of layered SNPs. (**a**) Calculated far-field emission patterns for a 1D dipole along the z-axis placed in a layer of cylindrical SNPs (blue solid lines) in a stack with different numbers of layers *N,* with surrounding medium *n* (outer left *N* = 1, center left *N* = 3, center right *N* = 11 with *n* = 1; outer right *N* = 11 with *n* = 1.5) and in an equivalent slab (red dotted lines). (**b**) Corresponding simulated near-field distributions of the dipole emission for the stack of SNPs (top row) and for the equivalent slab (bottom row).

To verify the validity of the approximation for the waveguided mode and the corresponding effective thickness, we calculated the emission by an oscillating 1D electrical dipole in the *x*-direction, in the center of a layer of cylinders with diameter 8 nm, with infinite extension in the *x*-direction and 101 cylinders in each layer (Figure 5). The 2D simulations were carried out in COMSOL Multiphysics for the nanoparticle layer and for a homogeneous thin film with the same dielectric constant and the same effective thickness (Figure S2). The results of the simulations are shown in Figure 5, for a monolayer of cylinders, and for stacks of three and eleven layers of cylinders. It can be seen that the far-field patterns (Figure 5a) and the near-field intensities (Figure 5b) for the nanoparticle layer and for the thin film are very similar, which validates the approximations. The simulations show that for the thicker layers, there is an important waveguiding effect. Due to reflections at the left and right edges of the SNP layer, there are standing waves formed in the layer.

The power coupled to the waveguided mode for an electrical dipole with random orientation and random position, for a homogeneous thin layer and for a layer of SNPs is, respectively, given by:

(7) $$L_{slab}=\frac{\omega^{4}p_{0}^{2}}{12\pi \varepsilon_{0}c^{3}}\cdot \frac{3\pi }{8}\left(n_{p}^{2}-n_{m}^{2}\right)kd$$

(8) $$L_{SNPlayer}=\frac{\omega^{4}p_{0}^{2}}{12\pi \varepsilon_{0}c^{3}}\cdot \frac{3\pi }{8}\left(n_{p}^{2}-n_{m}^{2}\right)\langle \beta_{∥}^{2}\rangle kd_{eff}$$

Note that the *β* factor is smaller than one, which means that for a layer of SNPs the coupling with the waveguide mode is less than for a continuous layer with the same effective thickness.

In many applications the embedding medium that surrounds the SNP layer is a polymer substrate (or another material) and above and below this substrate there is air. We are therefore particularly interested in the fraction of the light that is emitted from the layer into air. Usually the interface between the embedding medium *m* and air is parallel to the SNP layer. In LEDs or in photoluminescent layers, the fraction of light emitted into air should be as high as possible. In solar concentrators on the other hand, the aim is to trap light in a plate with a higher refractive index by total internal reflection (TIR). TIR in a medium with refractive index *n_m_* in contact with air takes place when the wave vector lies within a cone with half angle *θ_TIR_* = arcsin(1/*n_m_*). For randomly oriented dipoles that are homogeneously distributed over the SNP layer, the fraction of light that is emitted into air can be calculated from previous results (Supplementary Materials):

(9) $$\begin{array}{l} \eta_{air{,}_{RND}}=\frac{\langle \beta_{⊥}^{2}\rangle }{F}\left[1-\sqrt{1-\frac{1}{n_{m}^{2}}}\left(1+\frac{1}{2n_{m}^{2}}\right)\right]+\frac{2\langle \beta_{∥}^{2}\rangle }{F}\left[1-\sqrt{1-\frac{1}{n_{m}^{2}}}\left(1-\frac{1}{4n_{m}^{2}}\right)\right]\\ F=\langle \beta_{⊥}^{2}\rangle +2\langle \beta_{∥}^{2}\rangle +\frac{3\pi }{4n_{m}}\left(n_{p}^{2}-n_{m}^{2}\right)kd_{eff}\langle \beta_{∥}^{2}\rangle \end{array}$$

The third term in *F* represents the contribution due to waveguiding, and becomes negligible when the effective thickness is sufficiently small. Thin layers have a relatively high outcoupling fraction into air, as we take into account that $<\beta_{∥}^{2}>$ is typically larger than $<\beta_{⊥}^{2}>$ (Figure 3). When the layer is thicker, waveguiding becomes more prominent and less light is outcoupled to air (this is visible in Figure 5b).

**Figure 6:**
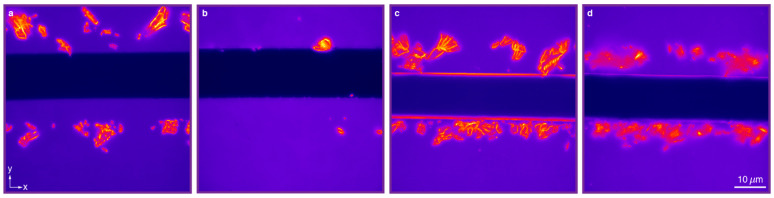
Overview of the fabricated samples. Fluorescence microscopy images of layered spherical SNPs with approximately one (**a**), three (**b**), eleven (**c**,**d**) layers deposited on top of a glass substrate, with embedding medium air (**a**–**c**) or an index matching fluid (**d**).

**Figure 7:**
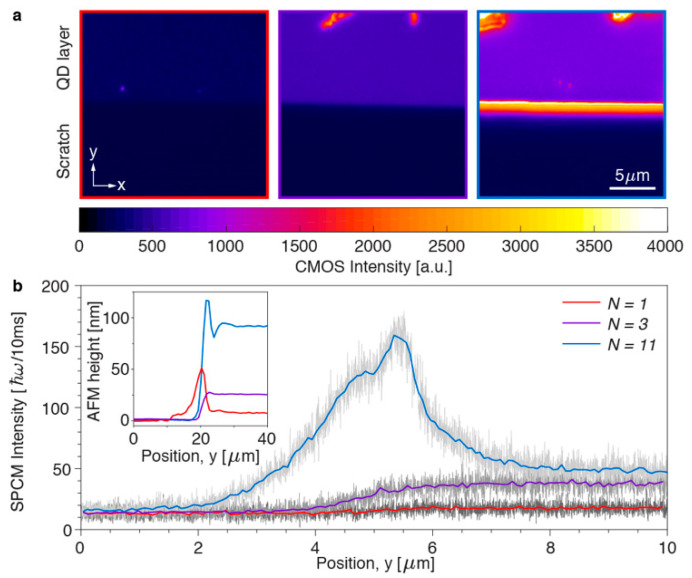
Measurement of the emission from a film of SNPs into air near an edge. (**a**) CMOS images of the fluorescence near the edge of a monolayer (left), a film with three layers (center) and a film with approximately eleven layers (right) of SNPs. (**b**) Single-photon counter measurement of the emission measured with a slit of 50 μm × 1 mm, corresponding to 0.5 μm on the substrate. Inset graph shows the atomic force microscopy (AFM) height profiles for the measured nanostructures.

In order to verify the dependency of outcoupling and waveguiding on the thickness of the SNP layer, we set up a series of experiments. First, we spin-coated layers of spherical core/shell CdSe/CdS quantum dots (*d_SNP_* = 8 nm) on glass substrates. The fabricated samples had film thicknesses of 8, 25 and 90 nm (Figure 1c,d), which correspond to one, three and eleven layers, respectively. The SNP films were scratched with a lancet to remove the quantum dots along a stripe, and to create a sharp edge of the deposited nanoparticle film as shown in Figure 6 (with bright field images in Figure S3). It can be seen that some aggregates of SNPs were deposited on the film along the scratch, during the removal process. The nanostructures were visualized by a microscope setup, in which the SNPs were excited with a blue LED and the photoluminescent emission was collected with a high-numerical aperture objective lens (NA 1.3, 100×). Spectral measurements show that only 15% of the LED light was absorbed in the 90 nm thick SNP layer, indicating that the excitation level is more or less the same for all SNPs. Some selected areas of interest are shown in Figure 7a, which depict the boundary of the SNP film with a minimal number of SNP clusters near the scratch. By averaging the pixel intensity over a horizontal line, we obtained a low-noise intensity profile of the emission near the edge of the SNP layer (Figure S4), where the SNPs have been removed in the region *y* < 5 μm. In addition, we also integrated the number of the emitted photons with a single-photon counting module (SPCM) through the slit aligned parallel to the scratch (Figure S5). This setup allows integration of the emitted photons for the same *y* coordinate during the slow movement of the sample in the *y*-direction with a nanometer-precision piezostage (Figure 7b).

The experimental results summarized in Figure 7 allow evaluation of the importance of photoluminescence outcoupling and waveguiding in SNP films with different thicknesses. In general, microscopy-based observation of waveguiding inside a planar structure is problematic, unless the waveguide is strongly scattering. Our approach enables direct detection of waveguided light at the edge of the nanostructure, where part of the light is scattered into the observation cone of the high NA objective. For a monolayer and for a film of three SNP layers, mainly light outcoupled from the plane of the structure (Figure 7b) is observed, with intensity at most 50 photons per 10 ms. In contrast to the thinner films, the 11-layer SNP film waveguides a lot of the emission towards the edges leading to a peak of 160 photons per 10 ms (Figure 7b). This peak is asymmetric, with different features on both sides of the edge: the left side (*y* < 5 μm) originates mainly from waveguided light and the right side is related to the peak of SNPs clustered at the boundary according to the AFM measurements in the inset of Figure 7b. Overall, the obtained experimental results are in agreement with our theoretical predictions (Equation (9), Figure 5a,b), revealing a strong dependence of photoluminescence waveguiding on the thickness of the SNP layer.

**Figure 8:**
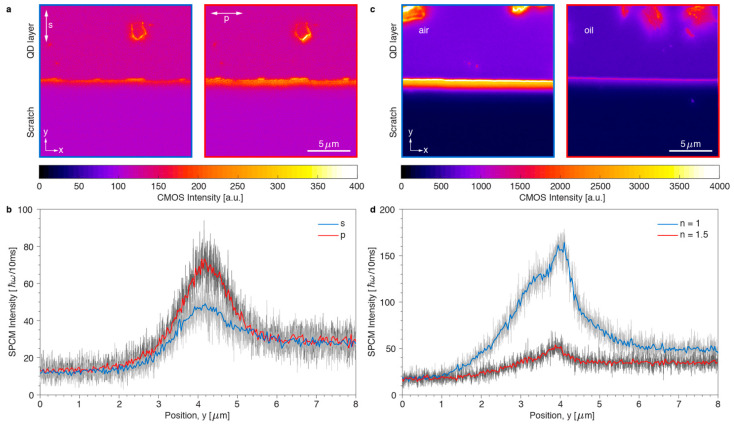
Experimental characterization of the emission waveguided within the thick film of SNPs (*N* = 11). (**a**) Fluorescent images of the sample with polarizer oriented perpendicular (left) and parallel (right) to the edge of the film. (**b**) Integrated single-photon intensity as a function of position for s- and p-polarized emission from the layer. (**c**) Fluorescence images of the edge of the film covered with air *n* = 1 (left) or covered with oil *n* = 1.5 (right). (**d**) Single-photon intensity profiles scanned over the edge of the film covered with air or oil.

Next, we characterized the properties of the photoluminescent light waveguided within the thick (*N* = 11) film of SNPs. By using a polarizer in the imaging path of the microscope, we found that the waveguided emission was mainly polarized with the electric field parallel to the edge of the film (Figure 8a). The dominance of p-polarized photons over s-polarization (Figure 8b) confirms that waveguiding is related to the *TE*_0_ polarized mode. Another important parameter for the waveguiding is the dielectric contrast between the SNP film and the surrounding medium. All measurements up to now were performed with SNPs deposited on glass substrates with air on top, leading to an asymmetric slab waveguide. To obtain a symmetric waveguide, we added a drop of oil immersion on top of the SNP film (Figure 8c), which had the same refractive index as the supporting glass (*n_m_* = 1.5). Due to the weaker dielectric contrast between the film (*ε_p_* = 6) and the overlaying medium at the edge of the waveguide (*ε_m_* = 2.25 instead of 1), scattering from the edge of the film into the observation cone of the objective lens was reduced (Figure 8d), as predicted by the simulations in Figure 8b. The observed properties of waveguided photoluminescence, polarization anisotropy and dielectric contrast-dependence, make thicker SNP films highly suitable for diverse waveguiding applications.

The presented theoretical and experimental results are important for the smart design of photonic devices based on SNPs. According to Equation (9), the estimated fraction of light outcoupled into air is 27% for a SNP monolayer immersed in a polymer, because waveguiding can be neglected. This factor decreases for increasing layer thickness, because more light is subjected to waveguiding in the SNP layer (the outcoupling factor reduces to 15.5% for *N* = 10 according to Equation (9)). This effect plays a crucial role for QD-based LEDs and organic light-emitting diodes (OLEDs), where the maximum outcoupling of photons into air is highly desired [1]. Indeed, recent reports show that LEDs attain the best emission characteristics when the thickness of the emitting layer does not exceed three layers of colloidal nanoparticles [22,23,24,25]. On the other hand, looking through the literature did not lead us to any other experimental evidence for waveguiding of light within the multilayer of SNP that is acting as the emitter itself. The thickness of the SNP layer may, apart from the optical properties, also affect the electrical properties (for example the efficiency of electron–hole recombination in the semiconductor [26]) and, hence, one should carefully tune and adjust the device architecture to find the optimal operating conditions. Based on the results obtained in this work, we claim that the effect of waveguiding in SNP layers should be taken into account in the design of SNP-based LEDs. The stronger waveguiding properties for thicker QD films, on the other hand, make them highly attractive for solar concentrators [27,28]. The amount of photoluminescence waveguided under TIR already reaches 74% for isotropically emitting spherical QDs that are suspended in a planar polymer slab [6]. The fraction of the waveguided light can be further increased to 84.5% (according to Equation (9), *η_air,RND_* decreases to 15.5%) by using a thick film with ten SNP layers embedded in a polymer medium (*n_m_* = 1.5). In this configuration, an important part of the light is waveguided in the SNP layer itself and this may lead to an increase of reabsorption events [4], which is an important loss mechanism in solar concentrators. A possible strategy to minimize reabsorption, while maintaining a large amount of total internal reflection in the polymer substrate, is to deposit the QD film onto a structured substrate with two height levels and a lateral dimension of, for example, 10 μm (smaller than the typical length related to reabsorption in the SNP waveguide). With this geometry an important amount of light is initially waveguided by the SNP film, and when it reaches the edge of a structure element, it is scattered into the substrate, in a direction that is still large enough to maintain total internal reflection in the substrate.

## 4. Summary and Conclusions

In conclusion, this work has demonstrated how the thickness of SNP films governs the emission of layered nanostructures. The presented theoretical framework shows that SNP monolayers have the highest outcoupling of emitted photons into the surrounding medium, whereas thick multilayered structures guide most of the photoluminescence towards the edges. These results have been confirmed with the set of experiments based on microscopic fluorescence imaging. The observed light phenomena for the layered SNP nanostructures unlock great potential to improve the efficiency and performance of diverse light-generating systems, ranging from LEDs and displays to solar concentrators and waveguides. 

## Data Availability

The data presented in this study are available upon reasonable request from the corresponding author.

## Supplementary Materials

The following are available online at https://www.mdpi.com/2079-4991/11/3/683/s1, Figure S1: Geometry, boundary conditions and meshing for numerical calculation, Figure S2: Absorption and effective thickness of packed nanorods, Figure S3: Bright field images of the scratch, Figure S4: Imaging through a slit for SPCM scanning, Figure S5: CMOS intensity profiles of the images in Figure 8, Video SI: waveguiding through the polarizer.
