# Supplementary material for: Waveguiding of Photoluminescence in a Layer of Semiconductor Nanoparticles
Source: Nanomaterials (Basel). 2021 Mar 9;11(3):683. doi: 10.3390/nano11030683 (PMC7999844; doi:10.3390/nano11030683)
Supplement: Supplementary file 1 [file nanomaterials-11-00683-s001.zip › supplementary materials/nanomaterials-1119994SI.pdf]

Waveguiding of Photoluminescence in a Layer of Semiconductor Nanoparticles

Yera Ye. Ussembayev [1,3], Natalia K. Zawacka [1,2,3], Filip Strubbe [1,3], Zeger Hens [2,3] and Kristiaan Neyts [1,3*]

[1] LCP research group, Ghent University, Technologiepark 126, 9052 Gent, Belgium; Yerzhan.Ussembayev@UGent.be (Ye.Ye.U.); NataliaKlaudia.Zawacka@UGent.be (N.K.Z.); Filip.Strubbe@UGent.be (F.S.)
[2] PCN research group, Ghent University, Krijgslaan 281, 9000 Gent, Belgium; Zeger.Hens@UGent.be
[3] Center for Nano and Biophotonics, Ghent University, Technologiepark 126, 9052 Gent, Belgium
* Correspondence: Kristiaan.Neyts@UGent.be

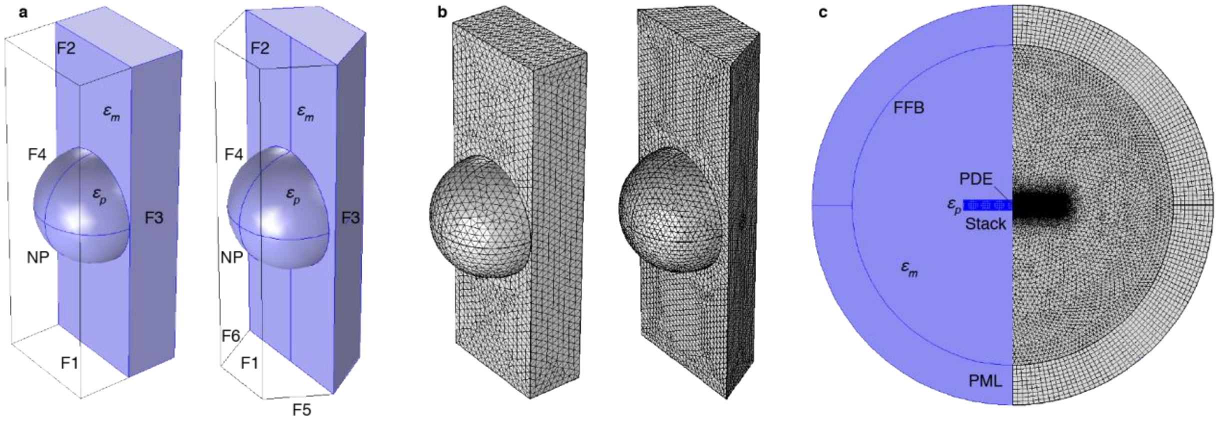

Figure S1. Geometry, boundary conditions and meshing for numerical calculation. **(a).** The designed geometry of the cubic (left) and hexagonal (right) periodic unit cells. **(b).** Meshing of the developed models. **(c).** Geometry and meshing of the model with a dipole emitter embedded withing the stack of nanoparticles.

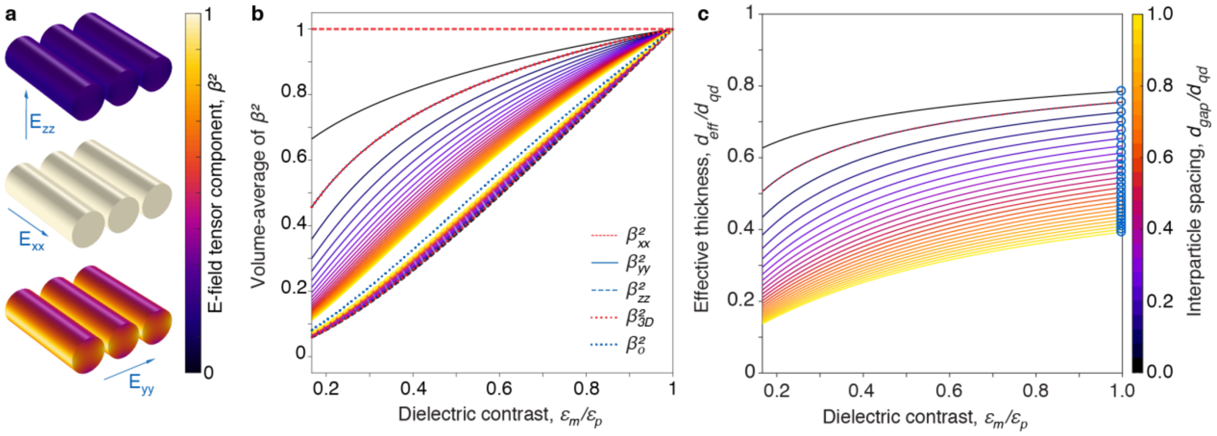

Figure S2. Absorption and effective thickness of packed nanorods. Distribution of the quadratic field factor β2(r) with the external field (blue arrow) perpendicular or parallel to the plane of the layer of nanorods **(a).** Volume-average <β2> **(b)** and effective thickness **(c)** as a function of dielectric contrast and interparticle spacing for nanorods

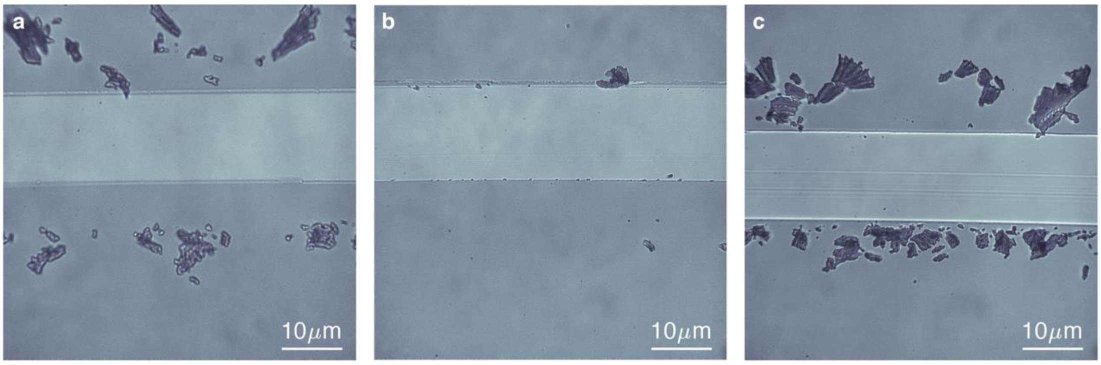

Figure S3. Bright field images of the scratch. The images correspond to the fluorescence images shown in Figure 6 (Main text) with a monolayer **(a)**, three layers **(b)** and eleven layers **(c)** of SNPs immersed in air.

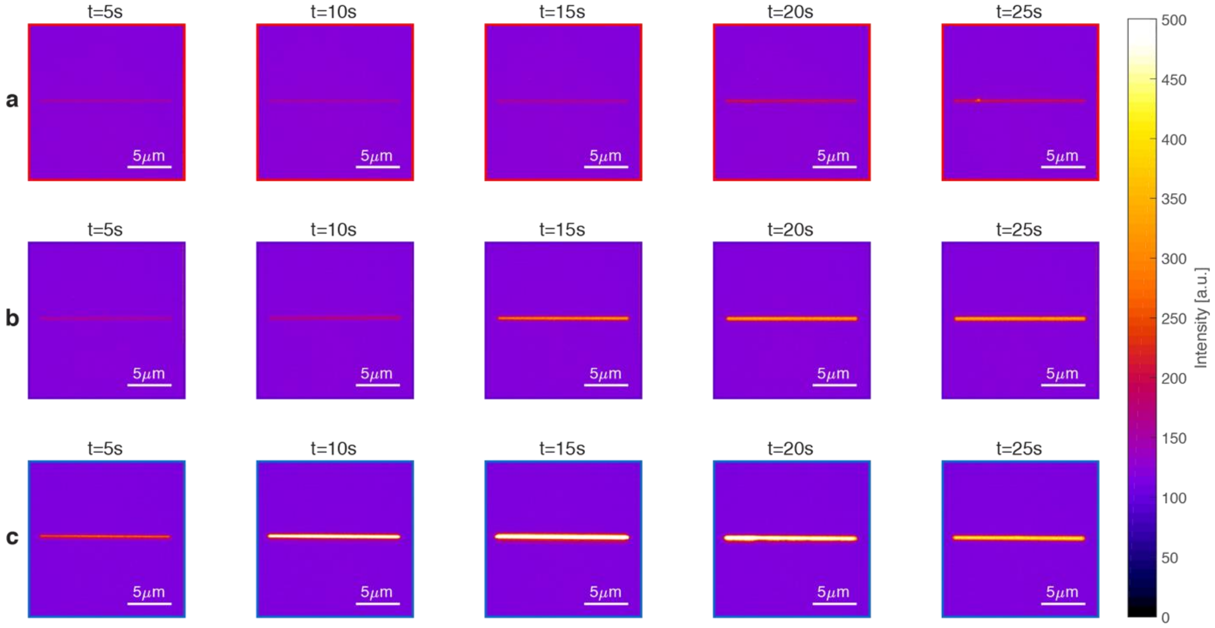

Figure S4. Imaging through a slit for SPCM scanning. The images show the time-step scanning over the scratch boundary through the slit for a monolayer **(a)**, three layers **(b)** and eleven layers **(c)** of SNPs.

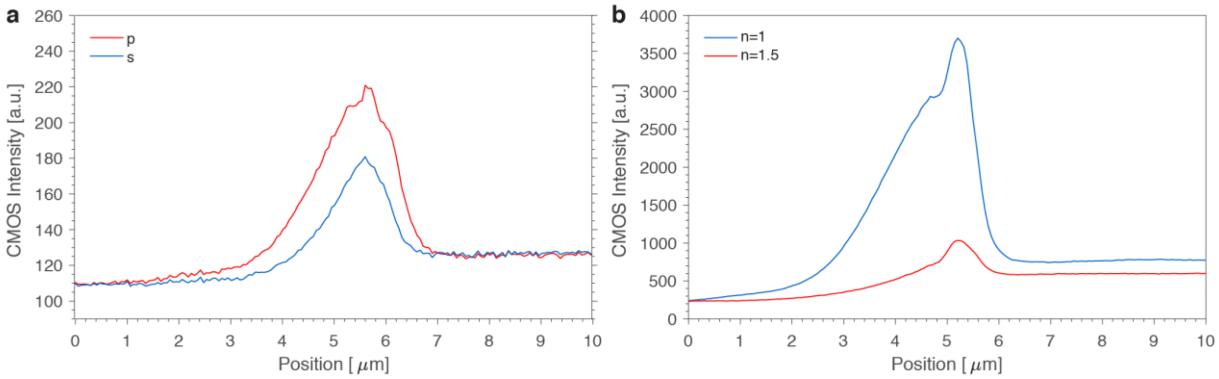

Figure S5. CMOS intensity profiles of the images in Figure 8 (Main text).

S1. Absorption for a Layer of Sub-Wavelength Size Particles

We consider a layer of particles on a grid with grid dimensions much smaller than the wavelength of light that is emitted or absorbed. The dielectric constant of the particles is ε_P and they are placed in a host medium with dielectric constant ε_m. We assume that the imaginary part of the dielectric constant is much smaller than the real part, which means that the absorption coefficient is small ($\mathrm{Im}(\varepsilon_P) \ll \mathrm{Re}(\varepsilon_P)$). We further assume that there are no free charges and that there is no permanent polarization in the particle.

Absorption of light is governed by the imaginary part of the dielectric constant. Because the imaginary part of the dielectric constant is small compared to the real part, we estimate the absorption based on the electric field in the material in the absence of absorption.

The electromagnetic field in a layer that is illuminated by monochromatic light can be calculated from the laws of Maxwell. Due to the fact that the SNPs have a diameter much smaller than the wavelength of light and that the layer of particles is continuous, we can assume that the electric field at a distance from the particles (further than the diameter of the particles, but closer than the wavelength) is in good approximation homogeneous. We further neglect scattering of light by the particles and diffraction effects. We call the homogeneous external field at some distance from the layer $\mathbf{E}_e$. The direction of this field $\mathbf{E}_e$ should correspond to the polarization of the incident light wave.

S2. Static Electric Field Calculations

If a layer of particles with real dielectric constant ε_P is placed in a static homogeneous external electric field $\mathbf{E}_e$ in the host medium ε_m, the field in and near the layer is distorted. The fields usually depend on the position coordinate $\mathbf{r}$ in the layer. The relation between the internal field vector in the layer $\mathbf{E}_i(\mathbf{r})$ and the far-field vector $\mathbf{E}_e$ is linear and is therefore described by a tensor β:

$$\mathbf{E}_i(\mathbf{r}) = \overline{\overline{\beta}}(\mathbf{r})\mathbf{E}_e \tag{1}$$

For a layer of particles, the tensor elements can be found by carrying out a static field calculation with a homogeneous external field as boundary condition. The cubic unit cell and the boundary conditions (DBC: Dirichlet Boundary Conditions and NBC: Neumann Boundary Conditions) are shown in Figure S1a. The meshing is illustrated in Figure S1b.

For a layer of particles the internal field is given by:

$$\begin{pmatrix} E_{ix} \\ E_{iy} \\ E_{iz} \end{pmatrix} = \begin{pmatrix} \beta_{xx} & 0 & 0 \\ 0 & \beta_{yy} & 0 \\ 0 & 0 & \beta_{zz} \end{pmatrix} \begin{pmatrix} E_{ex} \\ E_{ey} \\ E_{ez} \end{pmatrix} \tag{2}$$

We consider a monolayer of particles on a periodic square or triangular grid and calculate the fields in and near the layer with a static field calculation. The field can be perpendicular to the layer and there are several possibilities for the external field parallel to the layer.

S3. Absorption in a monolayer of nanoparticles

We consider the absorption of light in a layer of nanoparticle in the case that the imaginary part of the dielectric constant ($\mathrm{Im}(\varepsilon_P)$ is small. We assume that the particles are much smaller than the wavelength of light and much smaller than the absorption length associated with the imaginary part of ε_P. When a plane wave is incident on an infinite monolayer of particles, the amplitude of the oscillating electric field $\mathbf{E}_i$ in the layer is in good approximation given by equation (1), with $\mathbf{E}_e$ the amplitude of the oscillating field in the external medium, at some distance from the layer. The absorption in the layer per unit volume is given by:

$$\tfrac{1}{2}\omega\,\mathrm{Im}(\varepsilon_p)\left|\overline{\overline{\beta}}(\mathbf{r})\mathbf{E}_e\right|^2 \tag{3}$$

We assume that the spherical particles are arranged in a triangular or square grid. The absorption in the layer, for an external field along the layer normal (z-axis) or an external field parallel to the layer can be determined by averaging the absorption over a unit cell of the layer of particles:

$$\sigma_\perp = \sigma_{zz} = \tfrac{1}{2}\frac{\omega \operatorname{Im}(\varepsilon_p)}{c\sqrt{\varepsilon_m \varepsilon_0}} \int_{V_{unit}} \left(\beta_{xz}^2 + \beta_{yz}^2 + \beta_{zz}^2\right) dV = \tfrac{1}{2}\frac{\omega \operatorname{Im}(\varepsilon_p)}{c\sqrt{\varepsilon_m \varepsilon_0}} \left\langle \beta_\perp^2 \right\rangle$$

$$\sigma_{//} = \sigma_{xx} = \sigma_{yy} = \tfrac{1}{2}\frac{\omega \operatorname{Im}(\varepsilon_p)}{c\sqrt{\varepsilon_m \varepsilon_0}} \int_{V_{unit}} \left(\beta_{xx}^2 + \beta_{yx}^2 + \beta_{zx}^2\right) dV = \tfrac{1}{2}\frac{\omega \operatorname{Im}(\varepsilon_p)}{c\sqrt{\varepsilon_m \varepsilon_0}} \left\langle \beta_\parallel^2 \right\rangle \quad (4)$$

Note that due to the six-fold rotation symmetry (for the trigonal grid) or the four-fold rotation symmetry (for the quadratic grid), the absorption is independent of the aximuthal angle of the polarization of the light. The absorption anisotropy between polarization perpendicular and parallel to the layer is given by:

$$a_\sigma = \frac{\sigma_{//} - \sigma_\perp}{\sigma_{//} + \sigma_\perp} = \frac{\int_V \left(\beta_{//}^2 - \beta_\perp^2\right) dV}{\int_V \left(\beta_{//}^2 + \beta_\perp^2\right) dV} = \frac{\left\langle \beta_\parallel^2 \right\rangle - \left\langle \beta_\perp^2 \right\rangle}{\left\langle \beta_\parallel^2 \right\rangle + \left\langle \beta_\perp^2 \right\rangle}. \quad (5)$$

S4. Emission of light from a thin layer of nanoparticles

In many physical systems, the emission of a photon by a quantum-mechanical process is determined by an electrical dipole transition. In materials with a sufficiently high degree of symmetry, the orientation of the dipole moment that determines the emission of the photon is random. In this paragraph we assume that the dipole transition is located at a particular position in the nanoparticle layer and has random orientation. When a dipole emitter is embedded in a thin layer of nanoparticles, the emission has different contributions: light emitted above the layer, light emitted below the layer and light emitted into waveguided modes that are travelling along the layer. We will assume here that the nanoparticles are sufficiently small, so that scattering of the waveguided light can be neglected in a first approximation.

The field of a static electrical dipole $\mathbf{p}_i$ that is embedded in a nanoparticle layer in position $\mathbf{r}_i$, depends on the polarization charges that are created at the surface of the nanoparticle. At a distance much further than the size of the particle, the field in the external medium will also be a dipole field, but with another dipole moment $\mathbf{p}_e$. Reciprocity requires that the relation between the dipole moment $\mathbf{p}_i$ and the equivalent dipole $\mathbf{p}_e$ placed in a homogeneous external medium is based on the transposed tensor β^T:

$$\mathbf{p}_e = \overline{\overline{\beta}}^T(\mathbf{r}_i)\mathbf{p}_i \quad (6)$$

The equivalent external dipole moment $\mathbf{p}_e$ is in general not parallel to the internal dipole $\mathbf{p}_i$ and because the dielectric constant of the particle is larger, the amplitued p_e is smaller than the amplitude of the internal dipole moment p_i. The emission of the dipole $\mathbf{p}_i$ is equal to the emission of the equivalent oscillating dipole $\mathbf{p}_e$ that is embedded in the external medium with refractive index n_m. The total power emitted by the dipole in the layer is then given by:

$$L_p = \frac{\omega^4 n_m \left|\overline{\overline{\beta}}^T \mathbf{p}_i\right|^2}{12\pi\varepsilon_0 c^3}$$

When the orientation of the dipole $\mathbf{p}_i$ with amplitude p_i in the particle layer is random, the emission is given by averaging the previous formula over all directions in space of the dipole orientation.

$$L_{pRND} = \frac{\omega^4 n_m}{12\pi\varepsilon_0 c^3}\left(\left|\bar{\bar{\beta}}^T \mathbf{p}_i\right|^2\right)_{RND} = \frac{\omega^4 n_m}{12\pi\varepsilon_0 c^3}\frac{1}{3}\left[\beta_x^2 + \beta_y^2 + \beta_z^2\right] p_i^2 = \frac{\omega^4 n_m}{12\pi\varepsilon_0 c^3}\frac{1}{3}\left|\bar{\bar{\beta}}\right|_F^2 p_i^2$$

With F in the last expression referring to the Frobenius norm of the beta matrix. If the emission can take place from any point in the layer with the same probability, then the above value also has to be averaged over the coordinate **r** of the dipole emission. We then obtain:

$$\left\langle L_{pRND}\right\rangle = \frac{\omega^4 n_m}{12\pi\varepsilon_0 c^3}\frac{1}{3}\left[\left\langle \beta_x^2\right\rangle + \left\langle \beta_y^2\right\rangle + \left\langle \beta_z^2\right\rangle\right] p_i^2$$

For a layer with sufficient symmetry in the plane (trigonal or square lattice) the x and y averages are equal $\left\langle \beta_x^2\right\rangle = \left\langle \beta_y^2\right\rangle = \left\langle \beta_\parallel^2\right\rangle$ and correspond to emission of electrical dipoles that are parallel to the layer. The term $\left\langle \beta_z^2\right\rangle$ represents the contribution of the external dipole perpendicular to the layer. The emission has TE and TM contributions, with each contribution having its particular dependency on the angle θ with the layer normal:

$$P_{RND}^{TM} = \frac{\omega^4 n_m p_i^2}{12\pi\varepsilon_0 c^3}\frac{1}{8\pi}\left[\left\langle \beta_\perp^2\right\rangle \sin^2\theta + \left\langle \beta_\parallel^2\right\rangle \cos^2\theta\right]$$

$$P_{RND}^{TE} = \frac{\omega^4 n_m p_i^2}{12\pi\varepsilon_0 c^3}\frac{1}{8\pi}\left\langle \beta_\parallel^2\right\rangle$$

S5. Waveguiding in a thin layer of nanoparticles

The contribution of an emitting dipole to waveguided light in thin layer is estimated by analyzing coupling between the dipole and the dominant waveguided mode in a thin layer, which is the symmetric TE_0 mode with electric field parallel to the plane of the layer. For a sufficiently thin homogeneous planar film with thickness d_{film}, the electric field of the TE_0 mode decreases exponentially with the distance from the film, with decay constant $\alpha \approx \frac{1}{2}\left(n_p^2 - n_m^2\right)k^2 d_{film}$. The total power emitted in the waveguided mode for a dipole oriented parallel to the film is given by:

$$L_{wg,film} = \frac{\omega^4 p_i^2}{12\pi\varepsilon_0 c^3} . \frac{3\pi}{8}\left(n_p^2 - n_m^2\right) k d_{film}$$

which is small when the film is thin, because for a thin film the TE_0 mode is not well-confined.

For a thin layer of nanoparticles, the TE_0 mode in the embedding medium is (mainly) parallel to the plane of the layer and the field decreases exponentially with the distance from the layer. The decay constant depends on the excess polarization in a direction parallel to the layer (for example in the x-direction) of the TE_0 mode in the vicinity of the layer:

$$\alpha \approx \tfrac{1}{2} k^2 \frac{1}{S}\int_V \left(n^2 \beta_{xx} - n_m^2\right) dV .$$

This means that the formula for a thin film can be used by replacing the layer thickness by an equivalent thickness according to:

$$d_{eff} \approx \frac{1}{S}\int_V \frac{n^2 \beta_{xx} - n_m^2}{n_p^2 - n_m^2} dV .$$

For a layer of nanoparticles with random dipole orientation, homogeneously distributed over the layer, the power coupled to the TE_0 mode is given by:

$$L_{wg,SNPlayer} = \frac{\omega^4 p_i^2}{12\pi\varepsilon_0 c^3} \cdot \frac{\pi}{4} \left\langle \beta_x^2 \right\rangle \left(n_p^2 - n_m^2 \right) k d_{eff}$$

S6. Outcoupling efficiency to air for a nanoparticle layer

The average of the total emission of a dipole emitter with random orientation and random position is the sum of the emission into medium n_m and the waveguided light. This is given by the sum of the previous contributions:

$$L_{RND,total} = \frac{\omega^4 n_m p_i^2}{12\pi\varepsilon_0 c^3} \left(\frac{1}{3} \left\langle \beta_\perp^2 \right\rangle + \frac{2}{3} \left\langle \beta_\parallel^2 \right\rangle + \frac{\pi}{4 n_m} \left\langle \beta_\parallel^2 \right\rangle \left(n_p^2 - n_m^2 \right) k d_{eff} \right)$$

We consider a layer of SNPs embedded in a planar substrate with refractive index n_m and thickness much larger than the wavelength of light. The substrate is embedded in air on both sides. We now estimate the fraction of the light that is outcoupled from the planar substrate into air. This means that emission angle θ is either lower than θ_{TIR} or larger than π-θ_{TIR} with $\theta_{TIR} = \arcsin(1/n_m)$. To estimate this emission, we have to integrate the emitted power over the corresponding solid angle:

$$L_{RND,air} = \frac{\omega^4 n_m p_i^2}{12\pi\varepsilon_0 c^3} \cdot \left(\frac{1}{3} \left\langle \beta_\perp^2 \right\rangle \left[1 - \sqrt{1 - \frac{1}{n_m^2}} \left(1 + \frac{1}{2 n_m^2} \right) \right] + \frac{2}{3} \left\langle \beta_\parallel^2 \right\rangle \left[1 - \sqrt{1 - \frac{1}{n_m^2}} \left(1 - \frac{1}{4 n_m^2} \right) \right] \right)$$

The ratio of the emission into air over the total emission can be called the efficacy for coupling into air:

$$\eta_{air,_{RND}} = \frac{L_{RND,air}}{L_{RND,total}} = \frac{1}{3F} \left\langle \beta_\perp^2 \right\rangle \left[1 - \sqrt{1 - \frac{1}{n_m^2}} \left(1 + \frac{1}{2 n_m^2} \right) \right] + \frac{2}{3F} \left\langle \beta_\parallel^2 \right\rangle \left[1 - \sqrt{1 - \frac{1}{n_m^2}} \left(1 - \frac{1}{4 n_m^2} \right) \right]$$

$$F = \frac{1}{3} \left\langle \beta_\perp^2 \right\rangle + \frac{2}{3} \left\langle \beta_\parallel^2 \right\rangle + \frac{\pi}{4 n_m} \left\langle \beta_\parallel^2 \right\rangle \left(n_p^2 - n_m^2 \right) k d_{eff}$$

S7. Chemical synthesis of CdSe/CdS core/shell nanoparticles

CdSe/CdS core/shell quantum dots were synthesized by adapting the previously reported procedures [1–3], consisting in preparation of CdSe (core) QDs and subsequent growth of CdS (shell) material surrounding the initial particle. CdSe wurtzite particles were synthesized starting from CdO, N-tetradecylphosphonic acid (TDPA), oleyl alcohol (OlOH) and 10 g of trioctylphosphine oxide (TOPO), molar ratio Cd:TDPA:OlOH 1:6:16. The reaction mixture was heated for 1h at 150 °C under nitrogen atmosphere. The solution was then heated to 350 °C in order to dissolve CdO, then 2 mL of TOP was injected, followed by the injection of 2 M TOP-Se solution, Cd:Se molar ratio 1:2. The reaction time is limited to few seconds and stopped by a drop in temperature. The QDs were then precipitated from 20 mL of methanol and collected by centrifugation at 4000 rpm for 3 min. The supernatant was discarded and the QDs were again purified with toluene and methanol.

CdSe/CdS wurtzite particles were synthesized from CdO, oleic acid and 5 g of trioctylphosphine oxide; Cd:oleic acid molar ratio 1:5. The reaction mixture was heated for 1 h at 150 °C under nitrogen atmosphere. The solution was then heated to 350 °C in order to dissolve CdO, then 1 mL of trioctylphosphine was injected, followed by the injection of 2 mL of reaction solution. The reaction solution consisted of previously synthesized QDs seeds CdSe, 2.4 M TOP-S solution and additional TOP solvent; Cd:S molar ratio

1:1.2. After 5 minutes the reaction was quenched by a drop in temperature and the particles precipitated with 10 ml of methanol. The QDs were collected by centrifugation at 4000 rpm for 3 min and purified twice with toluene and methanol.

The QDs were dispersed and stored in toluene. The concentration of the wurtzite QDs dispersion was determined by their absorbance and the intrinsic absorption coefficients based on the Maxwell–Garnett effective medium theory [4,5].

The size of the CdSe particles was determined from the position of the first excitonic absorption peak using the sizing curve of Mulvaney et al [6], giving the diameter of 3.6 nm, while the size of the QDs was determined by TEM analysis, giving diameter of 7.4 nm (+/- 0.8 nm) for CdSe/CdS.

The quantum efficiency of the QDs solution was determined by an integrating sphere analysis via the two-measurement approach [7], giving 65%.

Bright field transmission electron microscopy (TEM) images were taken using a Cs corrected JEOL 2200 FS microscope. Absorption spectra were taken using Perkin Elmer Lambda 950 spectrometer.

Materials. CdO (≥ 99.99%), oleyl alcohol (85%) was purchased from Sigma-Aldrich. N-octadecylphosphonic acid (≥ 97%), N-tetradecylphosphonic acid (≥ 97%) were purchased from PlasmaChem GmbH. Trioctylphosphine (TOP, ≥ 97%) and sulfur (99.999%) were purchased from Strem Chemicals. Trioctylphosphine oxide was purchased from Merck Millipore. Selenium (200 mesh, 99.999%) and oleic acid (90%) from Alfa Aesar. The reaction solutions of TOP-Se (2 M) and TOP-S (2.4 M) were prepared by dissolving 1.56 g of the selenium powder and 0.78 g of sulfur in 10 mL of TOP respectively.

References

1. Cirillo, M.; Aubert, T.; Gomes, R.; Deun, R.V.; Emplit, P.; Biermann, A.; Lange, H.; Thomsen, C.; Brainis, E.; Hens, Z., "Flash" Synthesis of CdSe/CdS Core−Shell Quantum Dots. *Chem. Mater* **2014**, *26*, 1154–1160.
2. Carbone, L.; Nobile, C.; De Giorgi, M.; Sala, F.D.; Morello, G.; Pompa, P.; Hytch, M.; Snoeck, E.; Fiore, A.; Franchini, I.R.; Nadasan, M.; Silvestre, A.F.; Chiodo, L.; Kudera, S.; Cingolani, R.; Krahne, R.; Manna, L., Synthesis and Micrometer-Scale Assembly of Colloidal CdSe/CdS Nanorods Prepared by a Seeded Growth Approach. *Nano. Lett* **2007**, *7*, 2942–2950.
3. Drijvers,E.; De Roo, J.; Geiregat,P.; Fehér,K.; Hens,Z.; Aubert, T., Revisited Wurtzite CdSe Synthesis: A Gateway for the Versatile Flash Synthesis of Multishell Quantum Dots and Rods. *Chem. Mater* **2016**, 28, 7311-7323.
4. De Geyter, B.; Hens, Z., The Absorption Coefficient of PbSe/CdSe Core/Shell Colloidal Quantum Dots. *Appl. Phys. Lett* **2010**, *97*, 161908–161910.
5. Hens, Z.; Moreels, I., Light Absorption by Colloidal Semiconductor Quantum Dots. *J. Mater. Chem* **2012**, *22*, 10406–10415.
6. Jasieniak, J.; Smith, L.; Embden, J.V.; Mulvaney, P.; Califano, M., Re-Examination of the Size-Dependent Absorption Properties of CdSe Quantum Dots. *J. Phys. Chem. C* **2009**, *113*, 19468–19474.
7. Leyre, S.; Coutino-Gonzalez, E.; Joos, J.; Ryckaert, J.; Meuret, Y., Absolute Determination of Photoluminescence Quantum Efficiency Using an Integrating Sphere Setup. *Rev. Sci. Instrum* **2014**, *85*, 123115.
